# A Special Golden Curve in Human Upper Limbs' Length Proportion: A Functional Partition Which Is Different from Anatomy

**DOI:** 10.1155/2017/4158561

**Published:** 2017-01-23

**Authors:** Nan Wang, Jie Ma, Dan Jin, Bin Yu

**Affiliations:** ^1^Department of Orthopaedics and Traumatology, Nanfang Hospital, Southern Medical University, Guangzhou, China; ^2^Department of Pharmacy, The First Hospital of Jilin University, Changchun, China

## Abstract

*Aim*. The purpose of this study was to investigate the relationship between upper limbs' three functional partitions and the golden curve.* Materials and Methods*. We measured 30 subjects' right or left upper limb data and investigate the relationship between them and the golden curve by use of SPSS version 20.0 statistical software (SPSS, Inc., Chicago, Illinois), one-sample *t*-test.* Results*. There are four points on human's upper limbs which have no difference with the four points on the golden curve. And there is one point of which the difference is obvious. But we still could draw the conclusion that human upper limbs are accordant with the golden curve.* Conclusion*. Human upper limbs are accordant with the golden curve.

## 1. Introduction

The golden ratio (≈1.618), sometimes known as the golden section or golden number, has been fascinating philosophers, scientists, and artists for more than two millennia [1–4]. It appears in nature in a variety of forms, including the geometry of crystals, the spacing of stems in plants, and possibly the proportion of body parts in animals, and in the proportion of feature size in the human face and human body [5–9].

**Figure 1:**
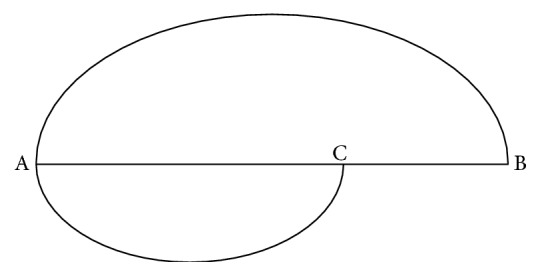
The straight line AB and the golden section point C.

Mathematically, the golden ratio designated by the Greek letter phi (Φ) is defined by relative lengths [1, 2]. If there is a point C which cuts a given straight line AB (Figure 1) to be a longer part AC and a shorter part CB and the ratio of AC to CB is equal to the proportion of the whole straight line AB to the longer part AC, the resulting ratio Φ is equal to 1.618⋯(Φ = |AC|/|CB| = |AB|/|AC| = 1.618⋯). In other words, if we name the ratio of the longer part AC to the whole straight line AB “*a*,” *a* = |AC|/|AB| = 1/Φ ≈ 0.618, and the ratio of the shorter part CB to the whole straight line AB = |CB|/|AB| = 1 − 1/Φ = 1 − *a* = *a*^2^ ≈ 0.382. Furthermore, if the length of the whole straight line |AB| is “1,” the longer part |AC| = *a*, and the shorter part |CB| = *a*^2^.

**Figure 2:**
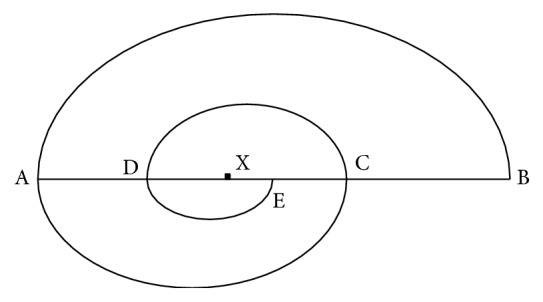
The straight line AB and the points C, D, E,…, X.

The point C is a golden section point of the straight line AB. Obviously, there are two golden section points on the line and C is only the right one. But how could we find the left one? If we are going to do this, firstly it is needed to find the golden section point D of the longer part AC (Figure 2). Secondly we need to find the golden section point E of the longer part CD. Furthermore we need to go on to find every golden section points on this line, on and on (infinitely many points in fact), until the final “end” point “X.” That is the left golden section point of this straight line AB. Thus we could get a curve BACDE,…, X which looks like a spiral line (it is not Fibonacci Spiral [10, 11]). In this “curve” |AC|/|AB| = *a*, |AD|/|AC| = *a*^2^, |AX|/|AB| = *a*^2^. There is much more to say about Φ mathematically. But we need to restrict our discussion to the human upper limb proportions.

**Figure 3:**
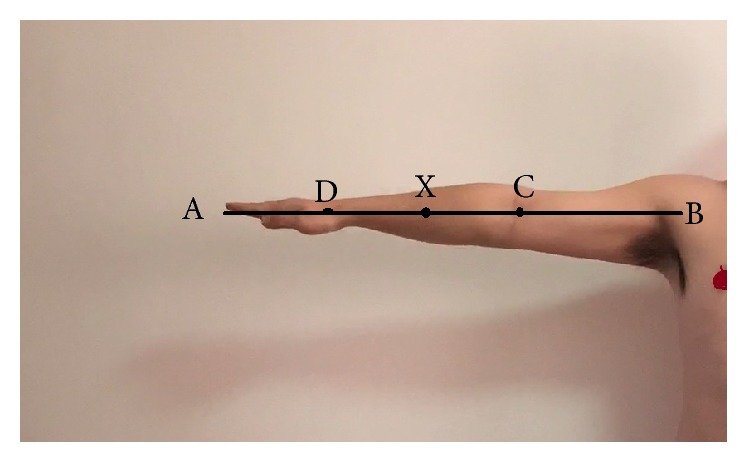
Human upper limb and the points A, B, C, D, E,…, X.

**Figure 4:**
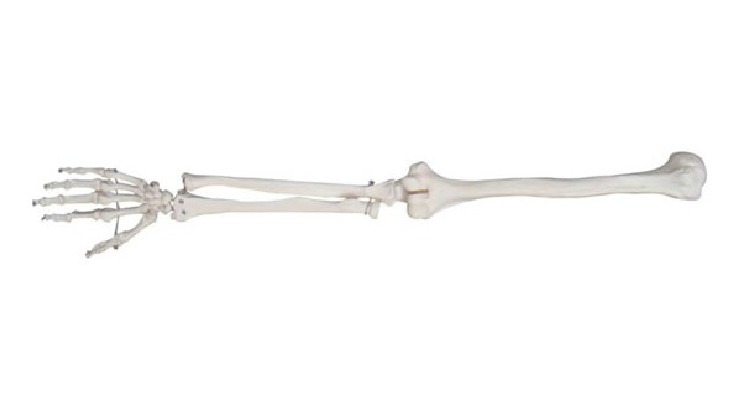
The bones of human upper limb.

Human upper limb [12] (Figure 3) is divided into three different parts: the upper arm, the forearm, and the hand. But according to anatomy, the forearm includes ulna and radius (Figure 4). Their lengths are not consistent and their proximal ends and distal ends are not in the same plane. Furthermore the carpal bones contain eight different parts, and the wrist joint is not strictly plane one. So it is not possible to mark off different parts of the upper limb from anatomy's point strictly just as some researchers had found that the relationship between human hand and the Fibonacci sequence is not supported mathematically [11]. But in functional view the upper limb could also be divided into three different parts: the upper arm is used to raise the whole limb, the forearm's function is to bend elbow and give the rotation needed to supinate and pronate the hand so as to reach something in different direction, and the hand is primarily used to hold something. In this paper, we investigated the relationship between human upper limbs' three functional partitions and the golden curve above.

## 2. Materials and Methods

### 2.1. Subjects

In this study we recruited 30 subjects. All of them are healthy adults without any upper or lower extremity disease or malformation. And they could not be over obesity or emaciated. They contained 12 male subjects and 18 female subjects. Each subject's single right or left upper limb data were measured and collected. We did not collect both left and right upper limbs data so as to exclude the impact due to the right and left upper limbs' length approximation. This study is part of the “healthy adults' harmonious gait analysis” research, and all the data collection were approved by the Medical Ethics Committee. Every subject had signed informed consent.

### 2.2. Data Measure and Collection

All the 30 adult subjects' single left or right upper limbs data were measured and collected. Every subject must be posing some fixed position which could represent extremities' function very well, without any movement while measuring is going on. Each set of data contains four parts.

**Figure 5:**
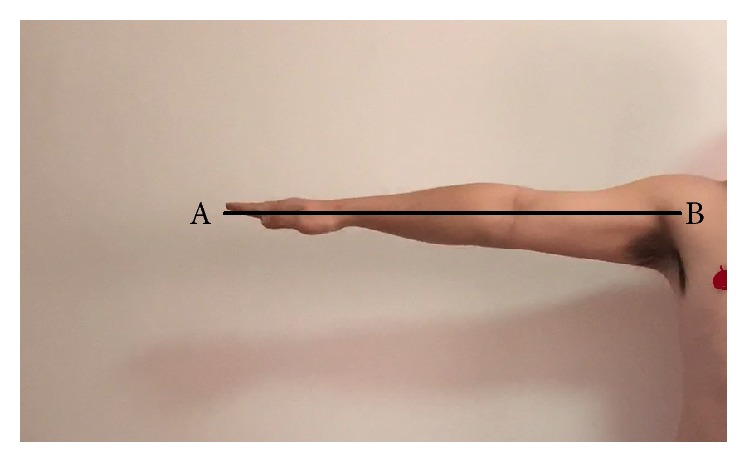
The method of measuring *l*1.

**Figure 6:**
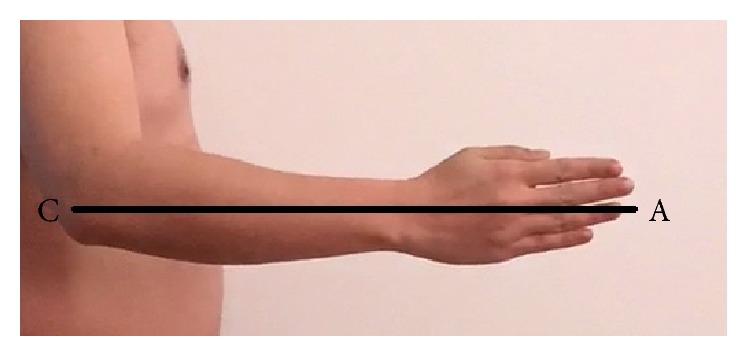
The method of measuring *l*2.

**Figure 7:**
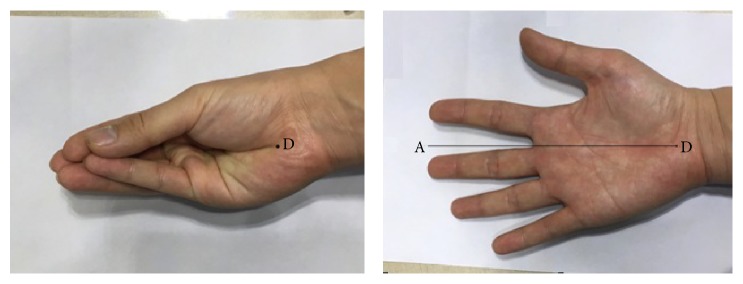
The method of measuring *l*3.

**Figure 8:**
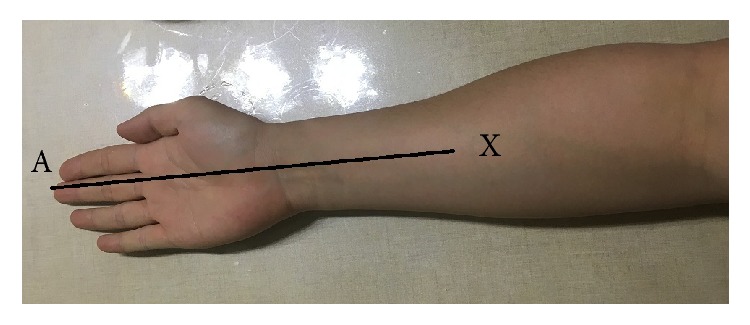
The method of measuring *l*4.

(1) First of all, every subject needs to outreach their upper limbs horizontally and straightly (Figure 5), and the distance from their shoulder joint (the joint top point on the upper surface) to the middle finger tip was measured and collected which is named “*l*1.”

(2) Secondly, every subject needs to bend their elbows while extending their wrists and hands at the same time (Figure 6). Then the distance from the back point of the elbow to the middle finger tip was measured and collected which is named “*l*2.”

(3) Thirdly, every subjects need to put their fingers together toward their palm just like holding something (Figure 7). And we marked the point D on the proximal end of their resultant longitudinal palmar crease which is obvious because of the thenar eminence and the hypothenar closing up. Then the subjects need to extend their wrist, hand, and fingers so as to measure the distance from the marked point D to the middle finger tip which is named “*l*3.”

(4) Finally, all the subjects extend their forearm, wrist, hand, and fingers straightly on the table (Figure 8). And we marked the point X on the forearm which is nearly the middle point of the forearms' transverse diameter where it is becoming smaller dramatically. Then we measured the distance from the marked point X to the middle finger tip which is named “*l*4.”

### 2.3. Statistical Analysis

The data analysis was performed by SPSS version 20.0 statistical software (SPSS, Inc., Chicago, Illinois), one-sample *t*-test. In this research we compare “*a*” (≈0.618) with the mean of *a*1  ( = *l*2/*l*1), “*a*^2^” (≈0.382) with the mean of *a*2  ( = *l*3/*l*2), and “*a*^2^” (≈0.382) with the mean of *a*3  ( = *l*4/*l*1), respectively. Statistical significance was set at *P* < 0.05. The intraobserver error is calculated by repeated measures, and the statistical significance was set at *P* < 0.05.

## 3. Results

(1) The means of *a*1, *a*2, and *a*3 are in Table 1 and the results of *t*-test are in Table 2.

From Tables 1 and 2 and Figure 11 we could see that comparing *a*1 with *a*  (≈0.618) by use of one-sample test, they have no significant difference. While comparing *a*2 with *a*^2^  (≈0.382) they have no significant difference either. But when comparing *a*3 with *a*^2^  (≈0.382) their difference is obvious.

(2) From Table 3 we could draw the conclusion that as the *P* value > 0.05, the repeated measurement values' differences were not statistically significant. That could be explained to be that the intraobserver error is very small and even insignificant statistically.

## 4. Discussion

**Figure 9:**
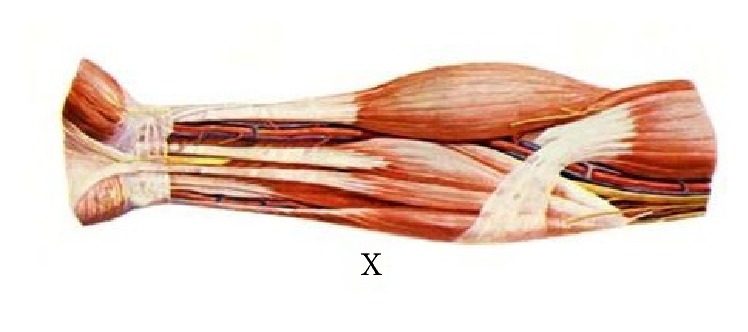
Forearm and X point.

**Figure 10:**
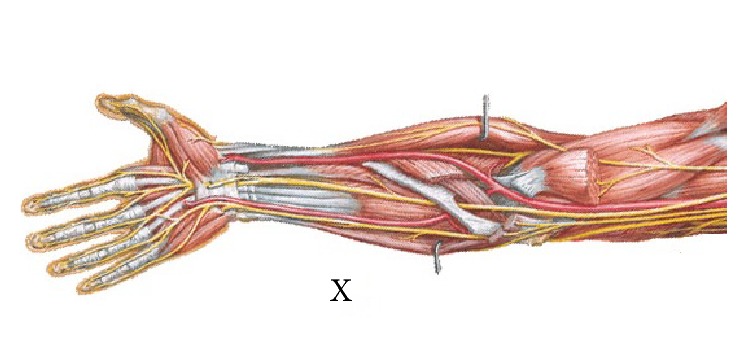
Human upper limb and X point.

**Figure 11:**
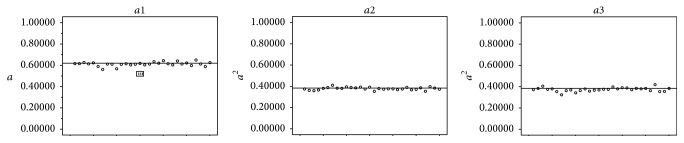
The difference between *a* and *a*1 is not obvious; the difference between *a*2 and *a*^2^ is not obvious either. The difference between *a*3 and *a*^2^ is obvious, but their values are close.

**Figure 12:**
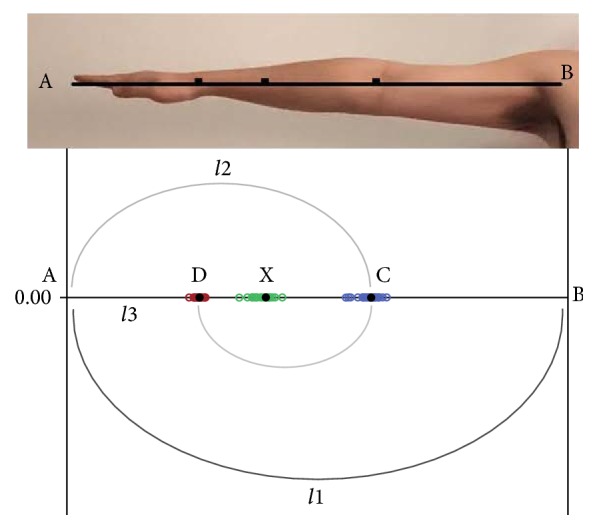
Human upper limb and golden curve.

(1) As the results in Tables 1 and 2 and Figure 11 showed, the difference between *a* and *a*1 is not obvious (*a*1 mean value is 0.6111014, *a*^2^ value is 0.618, and Sig.(2-tailed) is 0.057 (>0.05)), and the difference between *a*2 and *a*^2^ is not obvious either (*a*2 mean value is 0.3772133, *a*^2^ value is 0.382, and Sig.(2-tailed) is 0.053 (>0.05)). The difference between *a*3 and *a*^2^ is obvious, but their values are close (*a*3 mean value is 0.3734059, *a*^2^ value is 0.382, and Sig.(2-tailed) is 0.017 (<0.05)). Thus the four points ABCD (Figure 3) on human's upper limbs have no difference with the four points ABCD (Figure 2) on the golden curve (Figure 12). But the point X (Figures 2 and 3) on human upper limbs is different from the one on the curve although their locations are close (Figure 12). Even so, *a*1, *a*2, and *a*3 are nearly accordant with the characteristic of the golden curve introduced in Introduction, and we could also draw the conclusion that there are many functional partitions which are accordant with the golden section and the “golden curve” on human upper limbs.

(2) There are many studies showing that the golden ratio appears in the proportion of feature size in the human face and human body [9]. On the other hand some researchers believed that their coincidence cannot be proved from anatomy mathematically [10, 11]. In this research we proved that human upper limb is coincidence with golden ratio and the “golden curve” from the view of functional partition. Importantly, that is another way which is different from anatomy point. Human body contains many different systems, and there are nearly 20–30 different muscles and tendons on the forearm. So we believe the golden ratio and the “golden curve” is the comprehensive result of the complex organism rather than some single factor such as some muscle or cell. Therefore the result of Iosa and Fusco's research [8] could be understood easily. The human harmonious gait is accordant with golden ratio because the harmonious gait research is not from microscopic anatomy point but from a macroscopic view. From this point of view the “X” point is nearly the junction of many muscles and tendons of the whole human upper limb rather than some single junction of only one muscle and tendon. The macroscopic research method rather than microscopic view might be much more important than the conclusion of this research alone.

(3) In this research we found the “golden curve.” It looks like Fibonacci Spiral. But they are different in nature. In our opinion Fibonacci Spiral [11] is the golden section in the two dimensional world. There was some research about Fibonacci Spiral and human palm [11]. But the golden curve in our research is a method of looking for the points X in a straight line AB. And the human upper limb's length could be viewed as this line rather than a two dimensional plane. That is why human upper limb's length proportion is in coincidence with golden ratio and the “golden curve” rather than Fibonacci Spiral. Furthermore there is still some research to do so as to find out if this golden curve appears in other nature fields or in social world.

(4) Our study might contribute for medical and bionic research in the future. First of all, our study might contribute for medical, such as orthopaedics and plastic surgery, and bionic research in the future. For example, our research could help to evaluate upper extremity morphological and functional defect assessment for some patients with serious upper limb deformities because of injury, disease or congenital malformation, and even for some patients whose upper extremity defects were not very serious. Secondly, our research could help some doctors and their patients, who need to rebuild upper extremities, to design their surgical treatment, and to evaluate morphological and functional recovery better than before. Thirdly, after our future research of both upper and lower extremities, our research could be able to help normal people find underlying diseases in routine physical examination. For example, when we checking some children, teenagers or adults, if we find some part of their extremities such as the upper arm, crus or thigh disproportionality, maybe it is their humerus, tibia or femur epiphyseal growth has some problems. And in many cases, such patients' clinical symptoms might not be very obvious [13–15]. On the other hand, our research could help to design surgical treatment especially for some “healthy patients” who need plastic surgery. For example, in clinical there are some people who believe they are not tall enough. And, usually, they ask the doctors to extend their lower extremities or even both upper and lower extremities. In these cases our study could help them too. Furthermore, for some patients whose upper or lower extremity had already been lost and could not be rebuilt any more, our research could also offer help for prosthetic design and manufacturing.

(5) In this research we did not collect bilateral brachial data. That is because our subjects are different adults. The left upper limb data are nearly the same with the right ones'. And more importantly they would have bad impact on the degree of freedom in statistics and on the final results of our study.

(6) In our study there is one point X on human upper limbs of which the coincidence with golden curve is not obvious. X point is on the position where forearm's transverse diameter is becoming smaller dramatically. That is the place where forearm's muscle is becoming smaller and on the decrease. According to anatomy [12] this point is nearly the position where the muscle tissue is beginning to change into tendon tissue (Figure 9). From the view of the whole upper limb (Figure 10), X point is nearly demarcation point between muscle tissue and tendon tissue. And as the tissues different the diseases and medical methods are all different in some ways. While different people are at different muscular levels, it is possible that the X points on human upper limbs are in different positions. And it is not difficult to understand that the difference between the point X on human body and the point on the golden curve is obvious, although their positions are close indeed.

(7) On the other hand, the two “X” points' positions are very close indeed. From anatomy point, human's forearm contains many different muscles. And the junctions between most of them and the tendons are near the middle forearm. Although the forearm proximal end's transverse diameters differ due to the muscle variations there, the distal end does not vary dramatically because there are much more tendons rather than muscles. So we believe that the “X” point's position might be nearly immobile or variation in a narrow range in most human beings' forearms as it presenting the changing point. Furthermore, although “X” points vary in different subjects as a group, it is invariable in each one as a single person. In reality during our research we measured the “X” points exactly by all the members of our research team together. As we see eye to eye on the “X” point's position, we marked “X” point and measured “*l*4” together so as to make sure the “X” point's position being immobile on each subject's upper extremity and so the “*l*4” value being unchangeable either after repeated measurements.

(8) This study, of course, has some limitations. For example, we could improve our research method and measure different transverse diameter or circumference of forearm at different level such as every 5 millimeters. Obviously there would be much more work to do if we really improve our research method in the future. And at that time, “X” point might be measured much more accurately and might become in coincidence with “golden curve” mathematically—the result of which we did not prove today in this paper.

## 5. Conclusion

(1) Human upper limbs are accordant with the golden curve from the view of functional partition rather than anatomy zoning.

## Figures and Tables

**Table 1:** One-sample statistics. The mean of *a*1 ≈ 0.611, the mean of *a*2 ≈ 0.377, and the mean of *a*3 ≈ 0.373.

	*N*	Mean	SD	SE
*a*1	30	0.6111014	0.01909799	0.00348680

*a*2	30	0.3772133	0.01298421	0.00237058
*a*3	30	0.3734059	0.01850551	0.00337863

**(a):** 

	Test value = 0.618					
	*t*	df	Sig. (2-tailed)	Mean difference	95% confidence interval of the difference	
					Lower	Upper
*a*1	−1.978	29	0.057	−.00689862	−.0140299	.0002327

**(b):** 

	Test value = 0.382					
	*t*	df	Sig. (2-tailed)	Mean difference	95% confidence interval of the difference	
					Lower	Upper
*a*2	−2.019	29	0.053	−.00478665	−.0096350	.0000617
*a*3	−2.544	29	0.017	−.00859414	−.0155042	−.0016841

**Table 3:** Tests of within-subjects contrasts. The *P* values > 0.05; the repeated measurement values' differences were not statistically significant.

Measure: MEASURE_1									
Source	Times	Type III sum of squares	df	Mean square	*F*	Sig.	Partial Eta squared	Noncent. parameter	Observed power^a^
Times	Linear	.004	1	.004	.667	.416	.006	.667	.128
	Quadratic	.012	1	.012	2.571	.112	.022	2.571	.356
Times *∗* 1	Linear	.021	3	.007	1.111	.348	.028	3.333	.293
	Quadratic	.007	3	.002	.476	.699	.012	1.429	.144
Error (times)	Linear	.725	116	.006					
	Quadratic	.564	116	.005					

^a^Computed using alpha = .05.
